# Comparison of Light-Sheet Fluorescence Microscopy and Fast-Confocal Microscopy for Three-Dimensional Imaging of Cleared Mouse Brain

**DOI:** 10.3390/mps6060108

**Published:** 2023-11-10

**Authors:** Youngjae Ryu, Yoonju Kim, Sang-Joon Park, Sung Rae Kim, Hyung-Jun Kim, Chang Man Ha

**Affiliations:** 1 Research Strategy Office and Brain Research Core Facilities of Korea Brain Research Institute, Daegu 41062, Republic of Korea; ryj123@kbri.re.kr (Y.R.); pray4u96@kbri.re.kr (Y.K.); 2 Department of Histology, College of Veterinary Medicine, Kyungpook University, Daegu 41566, Republic of Korea; psj26@knu.ac.kr; 3 Dementia Research Group, Korea Brain Research Institute, Daegu 41062, Republic of Korea; herbtecn@kbri.re.kr (S.R.K.); kijang1@kbri.re.kr (H.-J.K.)

**Keywords:** light sheet fluorescence microscopy, fast confocal microscopy, three-dimensional mouse brain imaging, tissue clearing

## Abstract

Whole-brain imaging is important for understanding brain functions through deciphering tissue structures, neuronal circuits, and single-neuron tracing. Thus, many clearing methods have been developed to acquire whole-brain images or images of three-dimensional thick tissues. However, there are several limitations to imaging whole-brain volumes, including long image acquisition times, large volumes of data, and a long post-image process. Based on these limitations, many researchers are unsure about which light microscopy is most suitable for imaging thick tissues. Here, we compared fast-confocal microscopy with light-sheet fluorescence microscopy for whole-brain three-dimensional imaging, which can acquire images the fastest. To compare the two types of microscopies for large-volume imaging, we performed tissue clearing of a whole mouse brain, and changed the sample chamber and low- magnification objective lens and modified the sample holder of a light-sheet fluorescence microscope. We found out that light-sheet fluorescence microscopy using a 2.5× objective lens possesses several advantages, including saving time, large-volume image acquisitions, and high Z-resolution, over fast-confocal microscopy, which uses a 4× objective lens. Therefore, we suggest that light-sheet fluorescence microscopy is suitable for whole mouse brain imaging and for obtaining high-resolution three-dimensional images.

## 1. Introduction

Three-dimensional (3D) volumetric imaging is important for understanding the structural and functional characteristics of tissues and organs. The nervous system represents the most complex sample because individual neurons extend in long directions to many brain regions, and several neuronal groups compose neural circuits. Morphological studies on whole-brain imaging can explain the intrinsic and physiological brain functions by obtaining the population, altered morphology, and number of various cell types [1,2]. However, the brain is opaque, and light scattering, such as light absorption and autofluorescence, often occurs due to its complex organ structure [3]. Therefore, many tissue-clearing methods have been recently developed to acquire 3D tissue information, such as Scale, iDISCO, SeeDB, FocusClear, CUBIC, CLARITY, etc. [3,4,5,6]. These tissue-clearing techniques enable whole-brain imaging; however, imaging depth is restricted by light penetration and the objective-lens working distance. Furthermore, the tiling of image acquisitions is generally necessary owing to the limited field of view (FOV) of light microscopes, which can capture only a small portion of a specimen’s volume at a time [5]. The large volumes of image data that can be acquired from a cleared mouse brain must be processed using computational approaches, such as alignment and stitch, for each sequentially acquired image in order to reconstruct a 3D volume image for large-scale data.

Confocal microscopy produces 3D volumetric images with high signal-to-noise ratios through the reconstruction of a sectioned image of up to a couple hundred microns in size into cleared samples [7,8]. Thus, a disadvantage of confocal microscopy is that it is time-consuming due to its slow scanning process. Additionally, it is considered a slow process because it requires laser scanning of tissue sections that must be less than a hundred microns thick into small sections of a whole brain. Recently, high-speed and large-FOV confocal microscopes were developed to achieve three-dimensional and high-speed scan imaging of large tissues [9,10,11]. However, high-speed confocal microscopy has a photobleaching problem, and large-FOV confocal microscopy is not commercially available yet. Light-sheet fluorescence microscopy (LSFM) can acquire fast images of large-volume cleared mouse brains with reduced photobleaching because it is capable of capturing a single slice of a sample at a time using a light sheet [12]. Indeed, LSFM has been used for imaging in many tissue-clearing methods because it possesses a unique advantage in multi-scale volumetric imaging. Additionally, LSFM requires the reconstruction of tissue-sectioned images, and it has the limitation of detecting optical paths along the axis of the detection objective lens [13]. Both microscopy systems are not optimized for whole mouse brain imaging, even though light-scattering problems can be solved by tissue-clearing techniques. A high numerical aperture (NA) of the objective lens decreases the FOV and working distance for thick samples. A long working distance objective lens can be applied for obtaining a cleared thick brain image; however, it still has several disadvantages, such as a small FOV, a low signal-to-noise ratio due to axial scans across the brain to upright scans, and a long scan time for single-photon excitation [8,11]. Therefore, researchers need to select the most suitable microscopy for the deep imaging of thick tissues with low data analysis times, high-speed image acquisition, high-resolution imaging, and low 3D image rendering times.

In order to obtain a suitable 3D image of a cleared whole mouse brain, we compared the commercially available Dragonfly spinning-disk-type confocal microscopy (Andor Technology, Belfast, UK) with Lightsheet Z.1 (Carl Zeiss, Oberkochen, Germany), which was changed to a 2.5× objective lens and chamber, a modified sample holder of Lightsheet Z.1. This paper investigates the basic concept for suitable microscopy applications for whole mouse brain imaging and the most suitable way to acquire quick, wide, and deep images with high resolution.

## 2. Materials and Methods

### 2.1. Mice

The genetically modified Tg(Thy1-EGFP)MJrs/J mouse line was kindly provided by the laboratory of Dr. Rah in KBRI, which was originally purchased from Jackson Laboratories (JAX stock #007788, Bar Harbor, ME, USA) and bred in-house. The Tg (Thy1-EGFP) MJrs/J mice were genotyped with a primer set from the JAX protocol [14]. The genotyping procedure for the Thy1-EGFP mice was confirmed using the following primers: 5′-CGGTGGTGCAGATGAACTT-3′ and 5′-ACAGACACACACCCAGGACA-3′ for a 415bp internal positive control band; and 5′-GTAGGTGGAAATTCTAGCATCA-TCC-3′ and 5′-CTAGGCCACAGAATTG-AAAGATCT-3′ for a 324bp internal positive control band. For the sampling procedure, male and female mice between 6 and 8 weeks of age were deeply anesthetized with avertin [2.5%, tribromoethanol, intraperitoneal (i.p.)] in 1× PBS and transcardially perfused with ice-cold PBS to flush their blood vessels before 4% paraformaldehyde (PFA) in PBS was used. The fixed brains were postfixed with 4% PFA in PBS at 4 °C overnight. The mice were kept in groups of 2–5 animals per cage with ad libitum access to standard chow and water at a 12/12 light/dark cycle with “lights-on” at 07:00 at an ambient temperature of 20–22 °C and humidity of about 55% using a constant airflow. The wellbeing of the animals was monitored on a regular basis. The experimental design was reviewed and approved by the Institutional Animal Care Use Committee (IACUC) of the KBRI (IACUC-23-00016).

### 2.2. Whole Mouse Brain Clearing and Linear Expansion

A 4% PFA-fixed Thy-1-GFP mouse brain was cleared using the Rapid™ tissue-clearing method (Binaree Inc., Daegu, Republic of Korea). The procedure followed the manufacturer’s company protocol. Briefly, the fixed mouse brain was cleared into the Binaree Tissue-Clearing RapidTM Chamber, which is an electrophoresis chamber, for 4 h. Next, the cleared whole brain was transferred into a Binaree mounting solution and agitated at 50 rpm/37 °C for 24–36 h. Subsequently, the reflex index (RI) was matched to the 3D whole-brain image and imaged using Dragonfly spinning-disk-type confocal microscopy and Lightsheet Z.1 microscopy. The normalized expansion ratio of the initial state to the final distortion (at 36 h) was calculated for quantification.

### 2.3. Three-Dimensional Imaging Using Microscopy

We used the Lightsheet Z.1 (Carl Zeiss, Oberkochen, Germany) microscope from Zeiss for our imaging experiments. To enable whole mouse brain imaging, we applied an imaging chamber (Translucence Biosystems, Irvine, CA, USA) to a microscope with a 2.5× objective lens (Fluar 0.12 NA, Zeiss). The imaging chamber was approximately 42% larger than the chamber in the original Lightsheet Z.1 model. For the sample preparation, we glued a whole mouse brain to the bottom surface of the modified mounting holder. For the imaging process, we used a 2.5× objective lens (Carl Zeiss, Fluar, 0.12 NA) and a 5× illumination lens (0.1 NA, Zeiss) with a 488 nm excitation laser source (30 mW, Diode laser, Lasos laser GmbH, Jena, Germany). The data acquisition was performed using 10% laser power as the acquisition condition, and the camera (1920 × 1920 pixel resolution, PCO.Edge, Excelitas technologies, MA, USA) settings included an exposure time of 30 ms. Pixel size is 2.57 µm × 2.57 µm. All acquisitions were controlled by the ZEN software (Ver. 9.2.7.54, Carl Zeiss, Oberkochen, Germany). For the fast-confocal microscope, we used the Dragonfly 502 w model (Andor Technology, Belfast, UK). The objective lens used was a 4× lens (Nikon, Plan Apo λ 0.2 NA) with a 488 nm excitation laser source. (150 mW, Diode laser, Coherent, PA, USA). The data acquisition was performed using 10% laser power as the acquisition condition, and the camera (2048 × 2048 pixel resolution, sCMOS zyla, Andor Technology, UK) settings acquired an exposure time of 150 ms. Pixel size is 3.01 µm × 3.01 µm. All acquisitions were controlled by the Fusion software (Ver. 2.3.0.36, Andor Technology, Belfast, UK). The samples were imaged using an inverted microscope setup on a glass-bottom dish.

### 2.4. Modified Sample Holder

To address the limitations of the existing sample holder, which caused dead volume at the bottom of the sample due to the chamber structure and stage movement range of the Lightsheet Z.1 system, we designed a detachable cap using a J826™Prime 3D printer (Stratasys, Rehovot, Israel)). This cap matched the size of the dead volume, allowing for compatibility with the existing sample holder.

### 2.5. Stitching and 3D Rendering of the Stack Images

The images from both microscopies were rendered to 3D images using the Imaris software (ver. 9.2.1, Bitplane) and further analyzed. All files were converted into an ims format, and manual stitching was performed using the Imaris stitcher (ver. 9.2.1, Bitplane). Additionally, ImageJ was used for a sample expansion ratio analysis, and the Origin software (ver. 2020b, OriginLab) was used for histogram and Gaussian fitting. The workstation used for the analysis and post-processing had the following specifications: Intel Xeon^®^ Silver 4314 CPU 3.4 GHz, 128 GB RAM, Windows 10 x64 bit, and NVIDIA RTX A6000 GPU.

### 2.6. Measuring Spatial Resolution Using Fluorescent Beads

To verify the spatial resolution of the microscopes at low magnification, 4μm diameter fluorescent beads (#T7284, Invitrogen, Waltham, MA, USA) were utilized, and their FWHM of the point spread function was measured through Gaussian fitting. In the FCM case, measurements were taken by simply diluting the beads, applying them to a glass slide, and then measuring them. For LSFM, the beads were embedded in 2% agarose, fixed to sample mounts, and immersed in a matching solution for measurement.

## 3. Results

**Figure 1:**
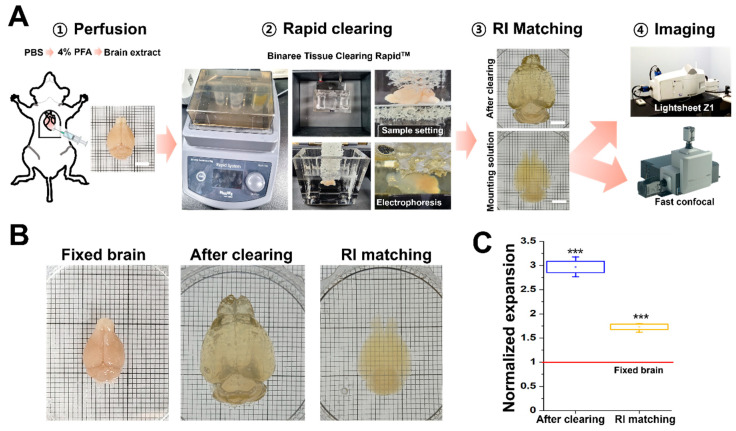
**Mouse brain sample clearing and preparation protocol.** (**A**) Experimental overview of large-scale imaging. Brain extracted after cardiac perfusion with 4% PFA and tissue clearing using the Binaree Tissue-Clearing Rapid^TM^ Chamber for 4 h, following the manufacturer’s protocol. The cleared whole mouse brain was imaged using FCM and LSFM. Scale bars: 5 mm. (**B**) Representative images illustrating the progression of the whole brain after fixation and extraction (fixed brain), after clearing (after clearing), and, finally, after 6 h of immersion in an RI matching solution (RI matching). The size of the cleared mouse brain underwent alterations with each preparatory step, followed by large-scale imaging of the RI-matched brain. (**C**) Statistical data showing alterations in expansion ratio following brain tissue clearing and RI matching. In comparison to the fixed brain, the cleared brain exhibited an approximate three-fold expansion. Following RI matching, a discernible trend of size reduction emerged, which remained 1.7 times larger than the original fixed brain (red line). n = 3 brains per group. *** *p* < 0.001 (unpaired *t* test vs. fixed brain). Data are normalized for the fixed brain and presented as means ± SEs.

To acquire optical 3D volume imaging of a specimen larger than a few centimeters, tissue-clearing methods are necessary. We used the Rapid™ tissue-clearing method (Binaree) to achieve transparency, which enabled imaging with light-sheet and fast-confocal microscopy. This method employed an electrophoresis step to accelerate the tissue-clearing process and allowed for the rapid clearing of whole mouse brains within a few hours. The cleared brains were placed in a Binaree-manufactured mounting solution, which is a high-RI matching solution (Figure 1A). During the tissue-clearing process using the Binaree system, the whole brain underwent expansion and shrank compared to its original size. Subsequently, we quantified the change in a sample volume at 12 h after immersion in the RI matching solution. The whole brain expanded approximately 3 times (2.97 ± 0.12) after the clearing process, and the cleared brain shrank around 1.7 times (1.73 ± 0.06) after immersion in the RI matching solution compared to the original fixed brain (Figure 1B,C).

**Figure 2:**
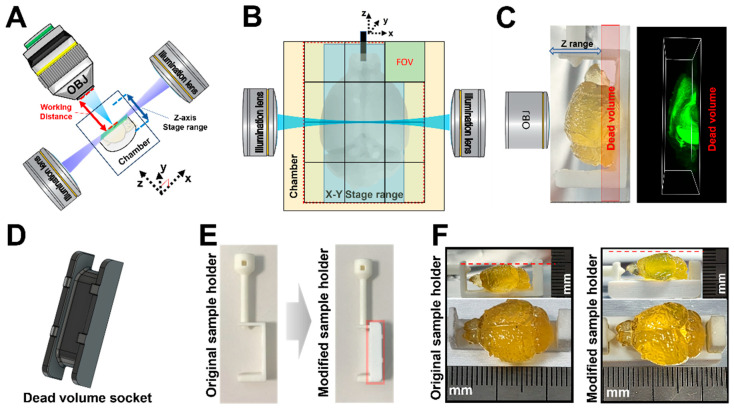
**Configuration of LSFM for whole mouse brain imaging.** (**A**) Layout of specimen imaging using LSFM. Despite the presence of a long-working-distance 2.5× objective lens (red arrow), LSFM had limitations in its Z-axis scanning capability due to restricted Z-axis stage movement range (blue arrow) and chamber size. (**B**) Representative FOV (4936 × 4936 ${µm}^{2}$) detection range for large samples using a 2.5× objective lens at 0.71× zoom in the light-sheet chamber. Pixel size: 2.57 µm × 2.57 µm). (**C**) Confined Z-axis imaging using the manufacturer’s sample holder. The dead volume in the imaging process occurred because the edge of the sample holder reached the imaging chamber. (**D**) Customized socket to reduce the dead volume. (**E**) A modified sample holder designed to minimize the dead volume. The customized socket was attached to the underside of the original sample holder (red box). (**F**) Comparison of top and side views upon mounting the whole mouse brain onto the original and modified sample holders, respectively.

In order to image a whole mouse brain, a system capable of imaging a wide FOV is required. Thus, researchers need to choose an image of appropriate resolution, identify the image from the whole brain, and consider the speed of image acquisition and data handling for multi-scale images. The spinning-disk-type confocal microscope is known for its relatively faster speed compared to traditional confocal laser-point scanning microscopes. Here, we used Dragonfly spinning-disk-type confocal microscopy as the fast-confocal microscope (FCM) and compared it with the LSFM. LSFM has the advantage of obtaining high-speed, large-scan images owing to its ability to acquire a single slice of sample at a time using a light sheet. Lightsheet Z.1 has a Z-axis limitation between the objective lens and the single light-sheet plane of the illumination lens (Figure 2A). Additionally, the XYZ-axis stage range in the imaging chamber size has limitations due to its large sample size and sample holder (Figure 2B). Thus, we changed the 2.5× objective lens of Zeiss and increased the inner volume of the imaging chamber of the Translucence Biosystems for whole-brain imaging. However, the original manufactured sample holder had a dead volume area for Z-axis imaging, which disrupted the movement of the objective lens (Figure 2C). To cover the dead volume, we created a socket that was attached to a sample holder, which caused the whole mouse brain to be positioned higher instead of at the end of the sample holder (Figure 2D–F).

**Figure 3:**
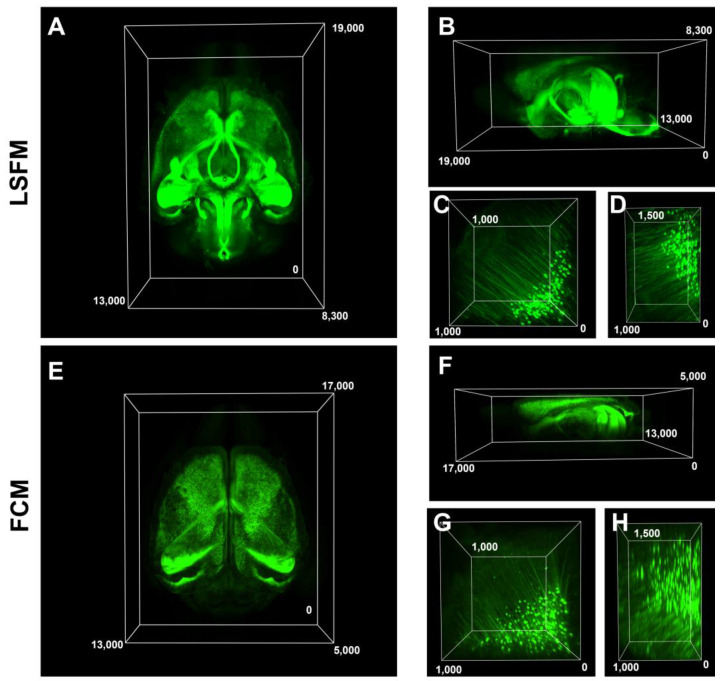
**Large-volume imaging of cleared whole mouse brain using FCM and LSFM.** A 2-month-old mouse exhibiting Thy1-EGFP expression in its brain cortex was sacrificed, followed by brain fixing, clearing, RI matching, and subsequent whole-brain imaging. Representations of the Thy1-EGFP whole mouse brain images (**A**,**E**) and views of the cortical region, including lateral (**B**,**F**), 3D XY (**C**,**G**), and 3D XZ (**D**,**H**) images acquired using indicated LSFM and FCM. Size units are in micrometers. Notably, FCM is incapable of acquiring whole-brain images, and the 3D XZ images were blurry compared to LSFM.

**Figure 4:**
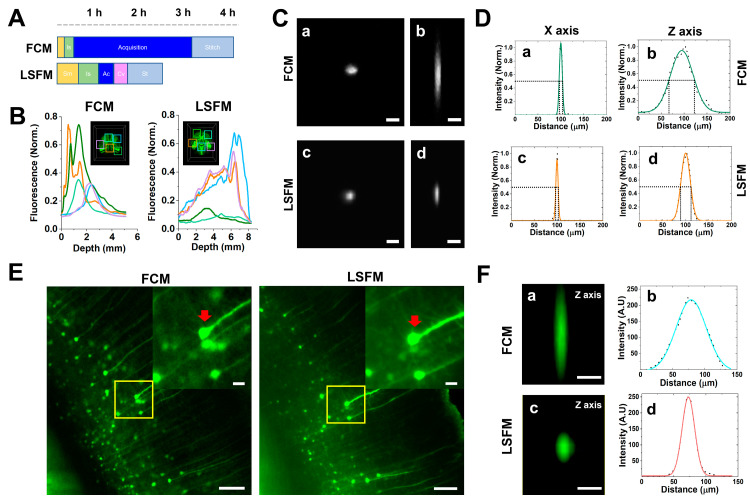
**Assessment of whole mouse brain imaging using both microscopes.** (**A**) Total time required for data acquisition, including the image-processing steps, such as specimen mounting (Sm), imaging setup (Is), acquisition (Ac), conversion (Cv), and stitching (St). (**B**) Fluorescent mean intensities with depth in various brain regions. (**C**) A comparison image of fluorescent microbeads in the X, Y (a, c), and Z axes (b, d) for the FCM and LSFM. Scale bar: 10 µm. (**D**) The image was analyzed using the full width half maximum (FWHM) intensity value for the fluorescent beads, and the relative analysis results for each image in panel (**C**) are represented. (**E**,**F**) A comparison image of neuronal cell bodies in the Z axis. The image represents the integrated intensity of same neurons using each microscope. Scale bar: 100 µm. Inset images (yellow boxes) are magnified. Scale bar: 20 µm. The elongated cell body image of the Z axis (red arrow) (a, c) and the FWHM analysis results for the intensity value for the neuronal cell bodies are represented in panels (**E**,**F**), respectively (b, d). Scale bar: 20 µm.

Subsequently, we compared the 3D volume images and resolution of both microscopes and identified that FCM had a limited imaging depth compared to LSFM, reaching around 3 mm deep before encountering difficulties in acquiring data, whereas LSFM enabled imaging of the entire brain (Figure 3A,B,E,F). The resolution of the large-volume images was relatively similar; however, the magnified images appeared blurrier in the FCM compared to the LSFM (Figure 3C,D,G,H). The volume imaging configurations of both microscopies for Figure 3 were included in Table 1. We further compared the image resolution, volume acquisition rate, and general characterization data of the 3D volume images in Table 2 using a single wavelength for each system described in Table 1. Using the FCM was quick and easy during the imaging process because it has an open chamber where the samples were fixed on a glass-bottom dish, and specimen mounting (Sm) and imaging setup (Is) were performed. In the LSFM case, the time it took to mount the sample, which included fixing it onto the sample holder and the stabilization of the RI matching solution, was relatively long, whereas the acquisition speed in volume per second was about 10 times faster than the FCM one (Table 2). We further calculated the total duration of the imaging acquisition (Ac), file conversion (Cv), and tile image stitching (St) process steps. The total process time for imaging a cleared mouse brain was approximately 4 h for FCM and 2.5 h for LSFM. (Figure 4A). The post-processing time as Cv and St significantly depended on the specifications of the analysis workstation. Thus, we used the same workstation specifications for post-processing with the Imaris software.

Although high-resolution 3D images are typically obtained using high-magnification FCM lenses, the wide-field imaging of large tissue areas is still time-consuming. By using low-magnification lenses to achieve a wider FOV, the Z-resolution is inevitably compromised. Most researchers are aware that confocal microscopy acquires high-resolution images with high signal-to-noise ratios using single-photon line scans. Thus, we performed a comparison between the images acquired via LSFM and FCM, and the fluorescent intensity with depth was acquired via LSFM (Figure 4B). We performed further experiments with fluorescent beads to confirm the lateral and axial resolutions of each system in Table 1. We calculated the full width half maximum (FWHM) of the intensity in the fluorescent beads, and the average FWHM values for the lateral resolution were similar to the 7.43 ± 0.03 µm for the FCM and 6.04 ± 0.02 µm for the LSFM, whereas the axial resolution was significantly increased to 58.15 ± 0.31 µm for the FCM and 23.29 ± 0.06 µm for the LSFM (Figure 4C,D). To verify that the axial resolution increased in the tissue-cleared mouse brain, we measured the FWHM values of the Z axis for the same neuronal cell bodies and found that it increased in the LSFM, with 55.37 ± 0.16 µm for FCM and 23.21 ± 0.04 µm for LSFM (Figure 4E,F). These results show that LSFM is more suitable for high-resolution 3D deep-brain images compared to FCM.

## 4. Discussion

In this study, for the first time, we compared FCM and LSFM for whole mouse brain imaging using a rapid tissue-clearing method by the Binaree Tissue-Clearing RapidTM system. As a comparison, we used the Dragonfly 502 w model as the spinning-disk-type FCM, which is known to be suitable for fast-scan imaging and large-volume imaging. Additionally, we applied the specifically modified Lightsheet Z.1 as the LSFM for whole-brain image acquisition by using an expanded chamber and low-magnification lenses for wide FOV acquisition. We also addressed the limitations of the specimen holder by designing a detachable socket, which allowed for imaging the whole mouse brain without a dead volume area.

FCM showed a significant difference in acquisition speed compared to LSFM, providing a 3/4 field of view (FOV) compared to LSFM but demonstrating convenience in the pre- and post-acquisition processes. On the other hand, LSFM required careful consideration of specimen exposure during the specimen mounting and potential damage from glue-chip formation and detachment. When comparing the image data from these two types of microscopes, it would have been ideal to use the same magnification as the 2.5× lens used in LSFM. However, we used the 4× lens for the confocal microscope because higher-resolution images could be obtained. Therefore, the total process time in Figure 4A depended on the magnification of the objective lens, the specification of the workstation, and the researchers’ skillful handling. Although long-working-distance objectives have been used in upright microscopy for whole mouse brain imaging [15], an upright confocal microscope requires a separate chamber for filling an RI matching solution and employing a specific deep long working distance dipping objective lens technique. Furthermore, the practical limitations of using special lenses suited for clearing samples, having long data acquisition times, and preventing the drying out of the RI matching solution by installing a special chamber in the microscope, have been considered. Recently, a spinning-disk-type upright confocal microscope was introduced to a very-high-speed serial-sectioning imaging system (FAST), which acquired high-resolution images of a whole mouse brain in a speed range comparable to that of light-sheet fluorescence microscopy. FAST is not commercially available and requires serial-section imaging using a microslicer and a Nipkow disk-based confocal scanner unit, such as block-face microscopy [16]. In this paper, we compared the commercially available FCM and LSFM and discussed their applications for brain imaging. Additionally, we discussed which microscopy is more efficient based on the specimen’s size and conditions. We compared each 3D volume image for whole mouse brain imaging with an FOV of 14.1 mm × 16.9 mm × 5 mm for FCM and an FOV of 13.2 mm × 18.9 mm × 8.3 mm for LSFM to the X-, Y-, and Z-axes, respectively. Although we compared the different image sizes and volumes, as shown in Table 1, we identified that LSFM has a unique advantage in multi-scale and 3D volume imaging compared with FCM.

In the case of FCM, a cleared brain with a thickness of up to approximately 500 μm can be easily viewed using a standard 10× lens, and high-resolution imaging is possible depending on the working distance of the lens. However, for samples with a thickness greater than 500 μm to up to a few millimeters, lower resolution with lower-magnification lenses might have to be applied.

The specimen mounting time of LSFM is essential and considered a critical factor in the imaging process, and with a specimen thickness ranging from hundreds of micrometers to approximately 2 mm, securing the sample with glue can be challenging. Thin samples, such as skin tissues, are challenging to image, particularly because simple glue fixation can lead to fluidity within the chamber solution [17]. Agarose embedding followed by sample mounting has been attempted to address this issue; however, this often results in sample folding, even for thin samples, requiring imaging over a wide range in three dimensions. Tissue-clearing methods are an important and crucial prerequisite for whole mouse brain imaging and microscope selection.

Numerous tissue-clearing techniques are available; however, we used the Binaree system, which removes whole mouse brain lipids by electrophoresis within a few hours. When we use this system, the whole brain was expanded approximately 2.5–3 times larger compared to its original size and shrinks to around 1.6–1.8 times after RI matching. Although the volumetric size of the whole mouse brain was magnified by the tissue-clearing method, LSFM took a high-resolution whole-brain image with an 8.3 mm Z-depth, whereas FCM took a half-brain image with a high-resolution that reached around 3 mm. Hence, researchers must choose a microscope system based on the size, thickness, and transparency of their sample. However, there is considerable confusion about whether there is an error in the tissue-clearing process or if there is a limitation with the microscope, and the problem can worsen if researchers assume that confocal microscopy automatically provides superior resolution or that LSFM is suitable for large-sample images.

In this study, we found that Lightsheet Z.1 is suitable for whole mouse brain imaging by using a modified objective lens, a solution chamber, and a sample holder socket compared to Dragonfly spinning-disk-type confocal microscopy. The modified Lightsheet Z.1 represents the one of advantage of Lightsheet 7 for large-volume imaging at a relatively low price. Taken together, these results provide useful information to researchers about the most suitable type of microscopy for large-volume images by considering factors such as entire specimen size, distribution and size of target proteins, acquisition time, and large data analysis.

## Figures and Tables

**Table 1:** **Comparison of configuration, data acquisition, and analysis conditions for both microscopes.** The data size for a large-volume image was analyzed with different FOVs, number of tiles, and Z-depths; however, the same Imaris software and workstation specifications were used.

Microscope	Mag.	FOV (mm)	Zoom	Z Depth (mm)	Tile (N × N)	Law Data Size (GB)	Convert & Stitch (GB)
**FCM**	**4×**	**3.1 × 3.1**	**1**	**5**	**5 × 6**	**68.4** (ims)	**36.6** (ims)
**LSFM**	**2.5×**	**4.5 × 2.8**	**0.71**	**8.3**	**3 × 7**	**106** (czi)	**60.8** (ims)

**Table 2:** **Characteristics’ data for both microscopes.** The acquisition speed and general characteristics for the imaging of cleared brain tissues with both systems are described.

Microscope	Acquisition Speed (Volume/Second)	Spatial Resolution (At Low Mag.)	Depth Imaging Range	Intermediate Medium	Specimen Mount	Cost
**FCM**	**Fast** (~ 0.14 ${mm}^{3}/s$)	**Low**	**Moderate** (High for LWD Obj.)	**Air** (MS for dipping Obj.)	**Easy**	**High**
**LSFM**	**Very fast** (~ 1.40 ${mm}^{3}/s$)	**Moderate**	**High**	**Ms** (RI:1.46~7)	**Tricky**	**High**

## Data Availability

All data is contained within the article. The authors can be contacted for any further information regarding the data within the article.

## Abbreviations

3D	Three-dimensional
Ac	Acquisition time
Cv	File conversion time
FOV	Field of view
FAST	High-speed serial-sectioning imaging system
FCM	Fast confocal microscope
FWHM	Full width half maximum
IACUC	Institutional Animal Care Use Committee
I.P.	Intraperitoneal
Is	Imaging setup
LSFM	Lightsheet fluorescence microscopy
NA	Numerical aperture
PFA	Paraformaldehyde
PFS	Perfect focus system
RI	Refractive index
St	Stitching processes time
Sm	Specimen mounting
WD	Working distance
Ms	Matching solution
